# Deep Learning and Geometric Modeling for 3D Reconstruction of Subsurface Utilities from GPR Data

**DOI:** 10.3390/s25206414

**Published:** 2025-10-17

**Authors:** Peyman Jafary, Davood Shojaei, Krista A. Ehinger

**Affiliations:** 1 Centre for Spatial Data Infrastructures and Land Administration, Department of Infrastructure Engineering, The University of Melbourne, Melbourne, VIC 3010, Australia; shojaeid@unimelb.edu.au; 2 School of Computing and Information Systems, The University of Melbourne, Melbourne, VIC 3010, Australia; kris.ehinger@unimelb.edu.au

**Keywords:** Ground Penetrating Radar (GPR), 3D utility mapping, keypoint detection, Mask R-CNN, YOLOv8, YOLOv11, DBSCAN, RANSAC

## Abstract

Accurate underground utility mapping remains a critical yet complex task in Ground Penetrating Radar (GPR) interpretation, essential to avoiding costly and dangerous excavation errors. This study presents a novel deep learning-based pipeline for 3D reconstruction of buried linear utilities from high-resolution GPR B-scan data. Three state-of-the-art models—YOLOv8, YOLOv11, and Mask R-CNN—were employed for both bounding box and keypoint detection of hyperbolic reflections, using a real-world GPR dataset. On the test set, Mask R-CNN achieved the highest keypoint F1-score (0.822) and bounding box F1-score (0.867), outperforming the YOLO models. Detected summit points were clustered using a 3D DBSCAN algorithm to approximate the spatial trajectories of buried utilities. RANSAC-based line fitting was then applied to each cluster, yielding an average RMSE of 0.06 across all fitted 3D paths. The key innovation of this hybrid model lies in its use of real-world data (avoiding synthetic augmentation), direct summit point detection (beyond bounding box analysis), and a geometric 3D reconstruction pipeline. This approach addresses key limitations in prior studies, including poor generalizability to complex real-world scenarios and the reliance on full 3D data volumes. Our method offers a more practical and scalable solution for subsurface utility mapping in real-world settings.

## 1. Introduction

Accurate mapping of underground utilities is crucial to prevent accidental damage during excavation, avoiding disruptions to essential services and minimizing repair costs [1]. This mapping is crucial for governments and residents, serving as a foundation for effective asset management, utility planning and construction safety [2]. For instance, in Australia alone, there are approximately 2400 asset strikes per year, leading to daily disruptions in the supply of essential services across communities and posing tangible risks to worker safety, potentially resulting in costly repairs [3].

Non-Destructive Testing (NDT) technologies like Ground Penetrating Radar (GPR) have demonstrated significant capabilities in furnishing valuable insights and data essential for the development of effective, safe and precise detection systems for buried objects [4]. GPR stands out as one of the most widely used near-surface geophysical methods for infrastructure imaging [5]. GPR instruments emit radio wave signals into a structure and capture echoes resulting from changes in material properties within it. Typically, the radio wave signal takes the form of a short pulse of electromagnetic energy. These signals, composed of coupled electric and magnetic fields, propagate into the material, scattering and reflecting off changes in its electric and magnetic properties. By analyzing the reflected signal strength and time difference of reception, GPR receivers lay the groundwork for imaging opaque structures [6]. GPR offers notable advantages, including the ability for easy data collection and a real-time interface display, facilitating prompt feedback on subsurface conditions. Additionally, GPR allows for centimeter-level image resolution adjustments by modifying its system bandwidth, enhancing precision in subsurface imaging [7].

However, the analysis of raw GPR signals often proves challenging, primarily due to the dominating influence of reflections from the upper layer, such as pavement. Furthermore, interpreting GPR data becomes more complex in the context of urban roads, marked by intricate geometries, fluctuations in moisture content, and the presence of unforeseen contaminants [8]. Accordingly, the process demands highly skilled specialists to make empirical decisions for recognizing underground objects amidst numerous natural and experimental noise sources within GPR data. This manual analysis of the full GPR data is not only arduous but also time-consuming. Consequently, there is a pressing need to develop fast and automatic underground utility recognition algorithms utilizing GPR data to streamline and enhance the efficiency of the analysis process [9].

A defining characteristic of underground objects in GPR images is the formation of hyperbolic reflection patterns, which arise from the curved travel paths of radar waves reflecting off buried targets. Accurately detecting and interpreting these hyperbolas is essential for identifying the position and depth of subsurface utilities. While recent studies have increasingly turned to deep learning models, especially CNNs, for hyperbola detection, many existing approaches remain constrained by several limitations. First, most studies focus solely on identifying bounding boxes rather than extracting precise summit points, which are critical for 3D localization. Second, many models are trained on synthetic datasets or limited real-world samples, raising concerns about generalizability in complex urban environments. Third, although some studies explore 3D reconstruction techniques, few provide an end-to-end pipeline for transitioning from detected hyperbolic features to accurate 3D mapping of linear utilities using real GPR data.

To address these challenges, this study presents a hybrid deep learning and geometric modeling framework for detecting, localizing and reconstructing underground linear utilities using real B-scan data collected from a multi-receiver GPR system. The aim is to overcome common bottlenecks in deep learning–based GPR analysis by combining high-precision keypoint detection with robust 3D mapping from real-world data, contributing a scalable and efficient solution for automated underground utility localization. This framework not only reduces the manual effort required for hyperbola interpretation but also enables accurate 3D mapping of subsurface infrastructure, offering a robust, scalable and data-driven alternative for GPR-based utility detection in complex urban settings.

The remainder of this paper is organized as follows. Section 2 provides background on the evolution of GPR data processing for civil structures, with a focus on recent advances in deep learning and 3D modeling for utility mapping and outlines key challenges in transitioning from 2D image analysis to 3D subsurface utility mapping. Section 3 describes the real-world GPR dataset used in this study, details the annotation process, and presents the proposed hybrid pipeline, including deep learning model architectures, keypoint clustering and 3D line fitting methods. Section 4 presents and discusses the experimental results, comparing model performance across detection tasks, visualizing 3D spatial patterns, evaluating the effectiveness of the geometric reconstruction process, and reflecting on the methodological innovations and broader implications of the work. Finally, Section 5 concludes the study by summarizing key findings, contributions and limitations, and outlines directions for future research in scalable, data-driven utility mapping.

## 2. Background and Literature Review

During the last few decades, GPR data has been employed for a wide range of tasks in civil engineering, from building, road and bridge inspection to geological and geotechnical applications, as well as underground utility mapping [6]. The effectiveness of these applications relies heavily on data processing techniques, which are central to extracting meaningful information from raw radar signals. Traditionally, such techniques have been grouped into signal-based methods and image-based methods [10]. In signal-based analysis, techniques such as amplitude interpretation, Fourier Transforms (FT) and filtering algorithms were used to interpret A-scans and reduce noise [11]. Conversely, image-processing and computer vision methods were developed to interpret B-scan radargrams. Preprocessing steps such as background removal, filtering and migration improved clarity, while algorithms like the Hough transform, template matching and edge detection enabled hyperbola identification for object localization in a variety of tasks [7]. While these traditional approaches laid the foundation for GPR interpretation, they rely on handcrafted features and often struggle in complex, noisy or heterogeneous environments [7,10,12]. The increasing demand for higher accuracy and automation in civil engineering has therefore driven a shift toward machine learning and more recently, deep learning techniques.

### 2.1. Utility Mapping Using GPR and Deep Learning

As Artificial Intelligence (AI) continues to advance, machine learning and deep learning techniques have attracted considerable attention due to their ability to deliver faster and more accurate results compared to image processing-based models [13]. Early studies in the field explored the use of Artificial Neural Networks (ANNs) for detecting regions of extended targets and integrating edge detection with other image processing techniques to localize buried objects [14,15]. Building on this, ANNs were employed to directly detect and localize buried pipes in GPR images by leveraging their characteristic hyperbolic signatures [16], as well as for direct analysis of hyperbolic features to localize steel reinforcement in concrete using Multi-Layer Perceptron (MLP) networks [17]. Pasolli, et al. [18] later proposed a four-step approach for the automatic detection and classification of underground objects, combining genetic algorithms for object detection with Support Vector Machines (SVMs) for material recognition. Birkenfeld [19] also utilized ANNs trained on hyperbolic reflection features, enhancing the detection of faint hyperbolas.

As in other vision-based analysis tasks across various fields of computer science and engineering, the analysis of GPR data has significantly advanced with the emergence of state-of-the-art deep learning techniques, particularly Convolutional Neural Networks (CNNs). These subsets of Deep Neural Networks (DNNs) are highly effective in image-related tasks due to their use of specialized convolutional layers, which automatically identify and learn spatial hierarchies of features within the data. By leveraging this hierarchical approach, CNNs can capture complex patterns, making them exceptionally proficient in applications such as image recognition, object detection, and image classification [20]. Besaw and Stimac [21] were among the first to apply CNNs to GPR data, using them to automatically extract features from 2D B-scans and classify buried explosive hazards, bypassing traditional feature engineering and achieving accurate threat detection through advanced deep learning techniques.

Advancements in deep learning have revolutionized GPR-based underground utility mapping by enhancing detection accuracy, reducing noise and enabling precise localization of subsurface infrastructure. Recent methodologies have focused on automating hyperbola detection, improving 3D mapping techniques and developing efficient end-to-end frameworks for underground utility localization.

Hyperbola detection remains central to identifying buried utilities in GPR B-scans. For example, Jaufer et al. [22] introduced a Faster R-CNN model that automates hyperbola detection, significantly reducing false positives and improving localization accuracy. This approach was validated on synthetic and real datasets, demonstrating its capability to streamline the mapping of buried pipes and support 3D utility visualization. Liu et al. [23] also proposed a YOLOv3-based framework that identifies hyperbolic pipeline reflections in GPR B-scans, followed by migration and iterative thresholding to pinpoint pipeline positions and depths. Their method achieved impressive results, with an average relative error of less than 4%.

Beyond hyperbola detection, several studies have explored integrating deep learning with advanced migration techniques for 3D mapping of underground utilities. Yamaguchi et al. [24] developed a 3D-CNN combined with Kirchhoff migration to classify and map transverse and longitudinal pipes. This approach improved detection accuracy over 2D-CNNs and successfully reconstructed precise 3D pipe maps validated through field experiments. Expanding on this, Lei et al. [25] introduced a hybrid framework that integrates RefineNet for clutter removal with reverse time migration (RTM) enhanced by total variation (TV) regularization. This method improves GPR image quality while reducing noise and artefacts, providing accurate localization and shape estimation of underground structures. Wang et al. [26] further contributed by employing radargram inversion using a DeepLabv3+ network to reconstruct permittivity maps directly from GPR data. Coupled with SLAM for geo-registration, their framework achieved high detection precision and enabled the seamless integration of subsurface maps with above-ground 3D environments.

Recent developments have also focused on creating end-to-end frameworks that simplify the utility mapping process. Su et al. [2] proposed the End-to-End underground utility localization (EUUL) model, inspired by the CenterNet, which features a lightweight CSPDarknet53 backbone for efficient feature extraction and an efficient channel attention (ECA) module to mitigate noise. Unlike traditional methods, the model employs a key point–regression mode to directly predict apex coordinates, bypassing the need for separate hyperbola detection and fitting. This approach achieved state-of-the-art performance with 97.01% localization accuracy, high inference speed, and improved robustness against noise. Collectively, these studies highlight the transformative potential of deep learning in GPR-based utility mapping, showcasing advancements in automation, precision, and efficiency for subsurface infrastructure detection.

### 2.2. Challenges in CNN-Based GPR Data Analysis

Despite the significant advancements in deep learning-based GPR data analysis, various challenges persist, limiting the full potential of these methodologies in practical applications. These challenges are rooted in data quality, model generalizability, computational efficiency, and the unique complexities of GPR data.

- Data availability and quality: A critical bottleneck in many studies is the lack of labeled GPR datasets, which limits the training and validation of deep learning models. Amaral Leila Carolina et al. [27] noted the reliance on synthetic data due to limited real-world datasets, which raises concerns about the generalizability of models in diverse field conditions. Models like YOLO and CenterNet achieved high accuracy [2,23], but their performance heavily relied on the availability of well-annotated training datasets. Augmentation techniques such as the use of synthetic radargrams or GAN-based enhancement are promising [26] but introduce new challenges in bridging the gap between synthetic and real-world data distributions. In addition, the quality of GPR data is often hampered by noise, clutter, and low-resolution imaging, which complicates the training and evaluation of deep learning models. Although [25] addressed clutter in GPR data through a customized RefineNet to preserve structural details while removing noise or Su et al. [2] introduced an ECA module to enhance feature robustness against noise in B-scans, the effectiveness of such solutions depends heavily on the diversity and realism of training data.
- Complexity of GPR data: The inherent complexity of GPR data, characterized by overlapping hyperbolic signatures and diverse subsurface conditions, poses challenges for accurate feature detection and classification. 3D-CNNs and integration of multiple scan types could be considered as a solution to capture spatial features and enhance performance of the models, but they may still struggle with data from highly heterogeneous environments and introduce additional computational overhead [9,24,28,29]. Due to such challenges, most existing studies, e.g., [22,23], often lack the direct use of advanced deep learning techniques for keypoint detection in detailed hyperbolic feature analysis, focusing instead on bounding box detection.
- Model generalizability and transferability: While CNNs excel in feature extraction, their ability to generalize across different environments and subsurface conditions remains challenging. Designing models that can detect varied object types in urban settings without extensive retraining is difficult, particularly when working with diverse subsurface conditions and utility types [30]. A notable challenge is reducing false positives caused by hyperbola generated from multiple reflections or environmental artefacts, which can significantly degrade model performance during field applications. This issue highlights the importance of developing robust detection frameworks capable of filtering such artefacts effectively [22]. Transfer learning methods can help mitigate this issue by leveraging pre-trained models on related datasets, though they require careful adaptation to the specific characteristics of GPR data [8,27,31].
- Computational and training demands: Deep learning models demand significant computational resources and time for training and inference. Barkataki et al. [32] optimized their CNN model for size estimation but required a powerful GPU to achieve acceptable training times. Additionally, Hou et al. [33] faced challenges in balancing detection accuracy and computational efficiency, particularly in tasks requiring simultaneous detection and segmentation with Mask R-CNN. The EUUL model by Su et al. [2] tackled this by employing a lightweight CSPDarknet53 backbone, achieving 125 frames per second (fps) inference speed. However, not all methods are as computationally efficient, particularly those using complex architectures like RefineNet or 3D-CNNs. Real-time deployment of CNN-based frameworks, such as those proposed by Zong et al. [30], is constrained by the need for high-speed processing without compromising accuracy. Ensuring scalability for large-scale surveys without compromising accuracy remains an ongoing challenge.

## 3. Materials and Methods

This study proposes a hybrid deep learning and geometric modeling framework for the automated detection and 3D mapping of underground utilities from GPR data. By “hybrid”, we refer to the integration of multiple methodological components, specifically, the combination of (1) deep learning-based keypoint detection, (2) unsupervised spatial clustering, and (3) model-based geometric fitting for 3D reconstruction. The framework is designed to address the previously discussed limitations in existing methods, such as reliance on synthetic data, the use of deep learning techniques for bounding box detection rather than keypoint detection due to the complexity of GPR data, and limited generalizability across different environments and subsurface conditions.

**Figure 1:**
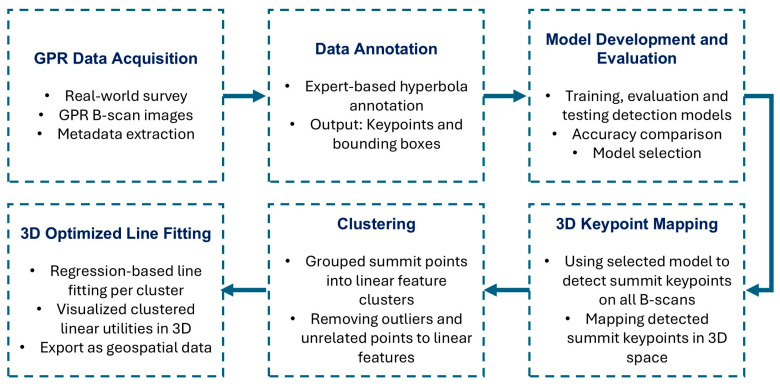
Workflow of the hybrid framework for automated detection and 3D mapping of underground utilities.

Our hybrid model first evaluates and compares the performance of three state-of-the-art models, YOLOv8, YOLOv11 and Mask R-CNN, for hyperbola summit point detection across parallel B-scans. The model with the highest accuracy is then used to extract summit keypoints, which are mapped into 3D space. These detected features are further processed through density-based clustering and regression pipelines to generate a 3D spatial representation of underground utilities. A flowchart of the proposed framework is presented in Figure 1. The following subsections provide detailed explanations of each component.

### 3.1. Data Collection

**Figure 2:**
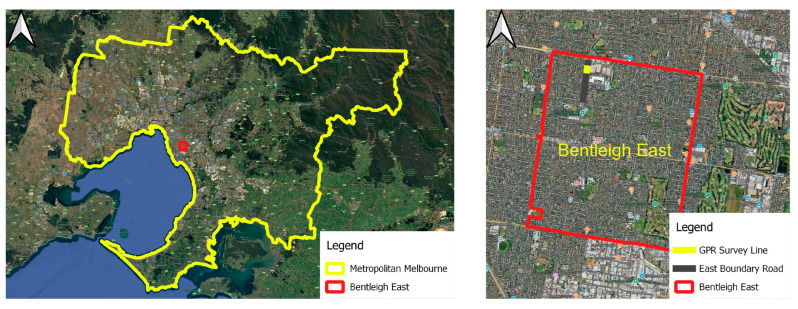
Location of the GPR survey conducted along East Boundary Road, Bentleigh East, Melbourne, Victoria, Australia.

The GPR survey was conducted along East Boundary Road in Bentleigh East, Melbourne, Victoria, Australia (Figure 2). The data were acquired using a multi-channel GPR system equipped with 20 receiver channels and a maximum penetration depth of 3.2 m. The raw survey data were stored in SEG-Y format and later processed using both R (version 4.4.0) and Python (version 3.11.5) programming environments. Metadata embedded in the SEG-Y textual header included key acquisition parameters:

− **Number of traces per line**: 322
− **Number of in-lines**: 20
− **Samples per trace**: 421

The raw SEG-Y comprised 421 samples per trace (vertical/time) and 322 traces per in-line (horizontal), i.e., a native B-scan of 421 × 322 pixels (px) per in-line. Post-processing then exported 20 parallel B-scan images at 15,054 × 3214 px, each representing a vertical subsurface slice aligned with the in-line trace direction. These very high-resolution images were produced by rendering the SEG-Y traces to amplitude radargrams using a custom preprocessing pipeline, which subsequently enabled more detailed and accurate manual annotation.

### 3.2. Manual Annotation of Hyperbolas

To generate high-quality training data for both bounding box and keypoint detection tasks, a custom graphical annotation tool was developed in R using the “imager” and “tcltk” libraries. This tool was used to manually annotate hyperbolic reflections in B-scan images by selecting three distinct keypoints for each visible hyperbola:

- Start (left leg-end);
- Summit (the apex or peak of the arc);
- End (right leg-end).

These keypoints define the geometry of each hyperbola and were chosen to reflect both the physical shape of radar reflections and the geometric consistency required for keypoint detection training.

Each hyperbola was defined visually based on curvature and intensity contrast, following expert geophysical interpretation practices rather than automated or physics-derived criteria. These annotations were performed under the guidance of a senior geophysicist who conducted field work at the study site and had intimate knowledge of the subsurface conditions. This ensured that ambiguous or overlapping arcs were correctly interpreted and excluded when not representative of valid buried features. Accordingly, the summit point was consistently identified at the maximum vertical elevation of the arc, and the Start/End points were selected at the inflection points where the curvature visibly diverged from the background. Hyperbolas that appeared partially truncated or merged with others were only annotated if a clear summit point and both ends were visually discernible.

**Figure 3:**
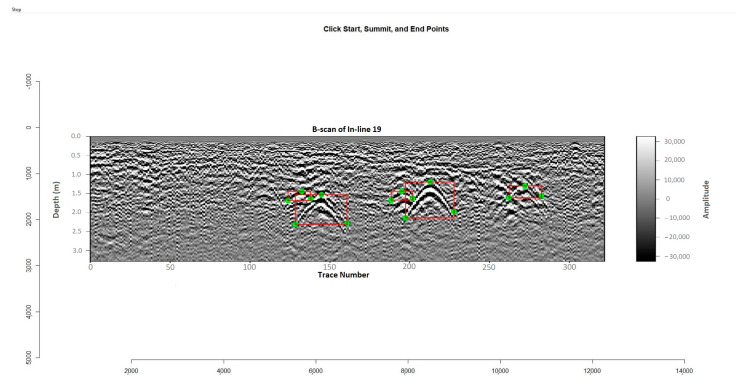
Example of manual hyperbola annotation using the custom R-based tool.

Each annotation was visually represented with green dots at the keypoints and a red bounding box surrounding the hyperbola (Figure 3). The annotations were exported into two CSV files:

− One containing the hyperbola ID and the X/Y coordinates of the three keypoints, along with their respective point types;
− Another is storing the minimum and maximum X and Y values for each hyperbola’s bounding box.

Although 20 parallel B-scan slices were extracted, each image contained multiple annotated hyperbolic targets with bounding boxes and summit keypoints. In total, 85 hyperbolas were manually labeled across the 20 B-scan images. Each hyperbola was annotated with three keypoints (Start, Summit, End) and an associated bounding box, forming the dataset used for model training and evaluation. This annotation strategy significantly increased the effective sample size, enabling the model to learn from over 85 instances of object–keypoint pairs while preserving real-world GPR complexity and ensuring expert-informed ground truth fidelity.

### 3.3. Deep Learning-Based Hyperbola Detection

To automate the detection of hyperbolic signatures in GPR B-scan images, this study employed three state-of-the-art deep learning models: YOLOv8, YOLOv11 and Mask R-CNN. The idea here is the extraction of the keypoints of hyperbolic features, and these models were selected based on their proven capabilities in object detection and pose/keypoint estimation tasks [34,35].

#### 3.3.1. YOLOv8 and YOLOv11 Keypoint Detection

YOLOv8 and YOLOv11, developed by Ultralytics, are the latest in the YOLO family and offer lightweight architectures, improved detection accuracy, and built-in support for keypoint detection tasks through pose estimation modules [36,37]. YOLOv8 introduces a streamlined architecture with decoupled heads and native keypoint detection capabilities through a direct regression approach, predicting keypoints directly instead of generating heatmaps, which are commonly used in early single-stage pose estimation methods [38]. This enables efficient training and inference on pose estimation tasks, especially in settings with sparse annotations or limited computational resources. Building upon YOLOv8, YOLOv11 incorporates architectural enhancements such as a stronger backbone, advanced feature aggregation through Cross-Stage Partial networks (CSP), and attention mechanisms to improve spatial awareness and feature representation. Both versions maintain a fully convolutional design and leverage Ultralytics’ modular pipeline, but YOLOv11 achieves superior accuracy and robustness across a wide range of pose benchmarks by integrating lightweight improvements and training strategies [39].

In this study, the annotated keypoints for each hyperbola were converted into the YOLO pose estimation format supported by both YOLOv8 and YOLOv11. These coordinates were normalized relative to image dimensions and paired with bounding box data for model training. Both YOLOv8 and YOLOv11 were initialized with pre-trained weights from the COCO dataset and subsequently fine-tuned on our domain-specific dataset of 20 real-world GPR B-scans. The models were trained using the Ultralytics framework with an image resolution of 640 × 640, and a maximum of 200 epochs. Early stopping was employed based on validation loss to prevent overfitting. Model performance was continuously monitored using precision, recall, F1-score, and mean Average Precision (mAP) metrics for both bounding box and keypoint tasks.

#### 3.3.2. Mask R-CNN with Keypoints

Unlike the YOLO family of models, Mask R-CNN is a two-stage detector that builds upon the Faster R-CNN framework by introducing an additional branch for pixel-level segmentation and optional keypoint estimation. The architecture consists of a backbone network (typically a ResNet-50 or ResNet-101) combined with a Feature Pyramid Network (FPN) to extract multi-scale features from the input image. In the first stage, a Region Proposal Network (RPN) identifies candidate object regions (Region of Interests, or RoIs). In the second stage, these RoIs are passed through RoIAlign, which preserves precise spatial alignment between input features and region proposals. The output is fed into three parallel branches: a classification head, a bounding box regression head, and a mask (segmentation) prediction head. For keypoint detection, an additional keypoint head, a fully convolutional subnetwork, is included to predict the locations of specific landmarks within each detected region [40].

For Mask R-CNN, the annotated hyperbola data were converted into COCO-style JSON format, incorporating bounding boxes and labeled keypoints. A custom dataset loader was implemented in PyTorch (version 2.4.0) to parse the annotation files and apply image transformations such as resizing and normalization. The Mask R-CNN architecture was built on a ResNet-50 backbone with Feature Pyramid Network (FPN) and modified to predict three keypoints (Start, Summit, End) in addition to bounding boxes. The annotations consistently preserve the left-right ordering, ensuring that the “Start” and “End” keypoints are semantically aligned across the dataset. Consequently, the model learns to differentiate “Start” and “End” based on their relative spatial positions. The model was trained using a multi-task loss function that included classification loss (cross-entropy), bounding box regression loss (smooth L1) and keypoint detection loss (L2 for visible keypoints). The model was fine-tuned with a single custom object class (‘hyperbola’) and three labeled keypoints (‘Start’, ‘Summit’, ‘End’). All COCO category mappings from the original weights were replaced, and the model was trained exclusively on our annotated dataset with no multi-class classification.

#### 3.3.3. Model Comparison and Selection

To support fair evaluation and tuning, splits were stratified by image, maintaining the relative density of hyperbolas per split. Accordingly, the dataset was partitioned at the image level into 15 B-scans for training, 2 for validation, and 3 for testing (20 total), with an approximately proportional distribution of annotated hyperbolas across splits to preserve class balance and scene variability.

To determine the most effective deep learning model for hyperbola keypoint detection, a comparative evaluation was conducted using three standard performance metrics: Precision, Recall and F1-score. These metrics were calculated for each model based on the predicted versus ground truth keypoints, aggregated across all annotated hyperbolas in the test dataset.

Precision measures the proportion of correctly identified keypoints among all keypoints predicted by the model. High precision indicates a low rate of false positives, which is essential for avoiding the misidentification of noise patterns or non-hyperbolic features as true summit points. Recall assesses the proportion of actual keypoints that were successfully detected by the model. High recall suggests robust detection sensitivity, which is critical for ensuring that all relevant hyperbolic reflections, especially weak or occluded ones, are captured. F1-score, the harmonic mean of precision and recall, provides a balanced metric for evaluating overall model performance, particularly when false negatives and false positives carry equal importance [41].

In addition to these metrics, we report mean Average Precision (mAP) for YOLO models, following the Ultralytics framework defaults. mAP@0.5 evaluates detection accuracy at a fixed Intersection over Union (IoU) threshold of 0.5, while mAP@[0.5:0.95] averages performance across thresholds from 0.5 to 0.95 in steps of 0.05 [42]. mAP condenses the precision–recall curve by calculating the average precision for each class, providing a single metric that reflects the balance between precision and recall across thresholds [43].

### 3.4. Three-Dimensional Summit Point Mapping

Based on the selected model, for each B-scan, summit points from detected hyperbolas were extracted. These were mapped to a 3D space using trace numbers (representing lateral position) and depth values from the annotated summit points. Key parameters included trace spacing between adjacent B-scans equal to 0.075 m and depth calibrated using image scale and SEG-Y metadata, equal to 3.2 m. The resulting dataset included the X (trace position), Y (vertical image coordinate), and Depth.

### 3.5. Clustering

Following the detection of summit keypoints from GPR B-scan images, a 3D density-based clustering approach was implemented to extract meaningful subsurface patterns from the detected keypoints. This step is essential because, in real-world GPR surveys, buried utilities rarely appear within a single B-scan; rather, they manifest as spatially coherent hyperbolas that extend across multiple parallel scans in the 3D space. While individual summit points may be accurately detected in isolation, without a mechanism to aggregate them into larger structures, their effectiveness in mapping continuous underground utilities remains limited.

Since underground utilities appear as continuous linear features across multiple GPR B-scans, clustering in three dimensions, trace number, depth and B-scan position, was critical to grouping corresponding summit points that likely belong to the same buried utility line. By clustering the points in three dimensions, the proposed method reconstructs the spatial continuity of features in a realistic survey environment. The 3D clustering process thus acts as a structural filter, allowing the framework to distinguish between isolated detections and those that align to form meaningful linear geometries, such as pipes or cables.

Due to the inherently noisy nature of GPR data and the spatial sparsity of detected hyperbolas across parallel scans, a robust 3D density-based clustering method was required to differentiate true utility traces from spurious detections. For this purpose, the Density-Based Spatial Clustering of Applications with Noise (DBSCAN) algorithm was chosen. According to [44], DBSCAN is particularly effective for geospatial data due to its ability to:

− Detect clusters of arbitrary shape in multi-dimensional space;
− Operate without requiring a predefined number of clusters;
− Identify and exclude noise points (outliers), which is especially important given the prevalence of false or incomplete hyperbola detections in GPR scans.

The 3D feature space construction in the previous step forms a 3D Euclidean space in which proximity reflects the spatial continuity of buried objects. To ensure isotropic scaling across all axes (i.e., avoid biasing clustering towards any one dimension), the data were standardized using z-score normalization prior to clustering.

### 3.6. Three-Dimensional Utility Line Fitting

Following the 3D clustering of summit keypoints, each cluster, representing a candidate underground linear feature, was processed through a model fitting pipeline to reconstruct the full and optimal spatial trajectory of utilities. This step aimed to approximate the spatial trajectory of buried infrastructure by transforming discrete summit points into smooth and continuous 3D lines that simulate the likely geometry of underground utilities.

Given the inherent disparity among detected summit points, caused by limitations in model accuracy, varying GPR signal quality, and complex subsurface conditions, these points rarely align to form a perfect geometric line in 3D space. Therefore, the objective of the fitting process is not to draw exact physical lines but rather to generate an optimized simulation of subsurface hyperbolic signatures, reconstructed in 3D. This approach allows for a spatially coherent representation of the most likely paths of underground utilities, extending the interpretation of hyperbolas beyond traditional 2D B-scan analysis into a comprehensive 3D context.

Two regression techniques were evaluated for this purpose: Total Least Squares (TLS) and RANdom SAmple Consensus (RANSAC). TLS, also known as orthogonal regression, minimizes the perpendicular distances from all points to the fitted line, offering a geometrically optimal solution in 3D space [45]. This was implemented using Principal Component Analysis (PCA), where the first principal component defines the best-fit line through the data. RANSAC, on the other hand, was selected for its robustness to noise and outliers. It fits a linear model by repeatedly sampling random subsets of the data, identifying the model with the most inliers based on a residual threshold [46].

For each cluster, both models were fitted using the 3D coordinates: Trace Number (X), B-scan-derived position (Y) and Depth (Z). Root Mean Square Error (RMSE) was calculated for each model for model comparison and selection.

The resulting fitted lines were stored as 3D polylines and exported as geospatial features, forming the basis for further mapping, visualization and integration with GIS tools.

## 4. Results and Discussions

This section presents the training configurations, model performance metrics, clustering outcomes, and post-clustering spatial reconstruction results. We evaluated three detection models—YOLOv8, YOLOv11, and Mask R-CNN—on a dataset of high-resolution B-scan images (15,054 × 3214 px), using both bounding box and keypoint detection tasks. Each model was trained using appropriate architecture-specific hyperparameters and evaluated based on bounding box and pose detection precision, recall and F1-score.

### 4.1. Hyperbola Detection

All models were initially set to train for up to 200 epochs; however, early stopping based on validation loss was applied, resulting in different actual training durations for each model. YOLO models used resized 640 × 640 inputs with the Ultralytics framework, while Mask R-CNN was trained on original-sized images using the PyTorch framework. For YOLO models, the original B-scan images were automatically resized to 640 × 640 px, following Ultralytics’ training framework requirements. This resizing ensured compatibility with the YOLO input format while preserving the relative spatial geometry of annotated keypoints and bounding boxes. Training times, model sizes and optimizer configurations are summarized in Table 1. It is important to note that Table 1 presents the training configurations of YOLO and Mask R-CNN models side by side for documentation purposes only; due to architectural differences, these hyperparameters are not directly comparable and were selected to suit each model’s implementation framework. Table 2 also summarizes the detection and keypoint estimation performance of the three models.

#### 4.1.1. Training Behavior and Efficiency

**Figure 4:**
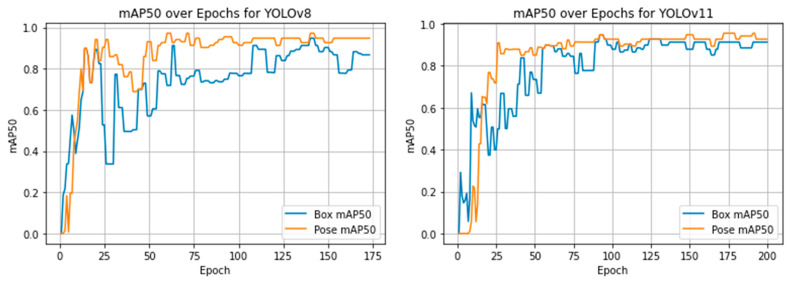
Training progression of YOLOv8 and YOLOv11 based on mAP@0.5 over epochs.

**Figure 5:**
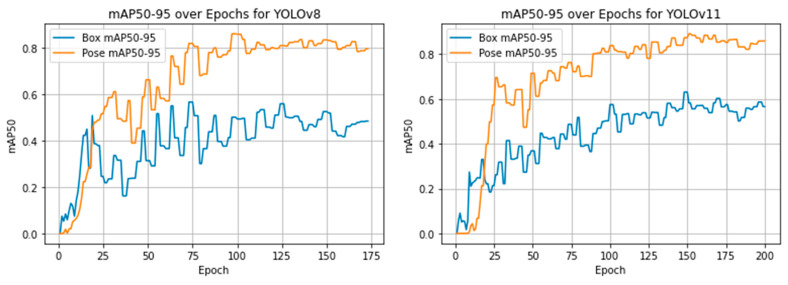
Training progression of YOLOv8 and YOLOv11 based on mAP@[0.5:0.95] over epochs.

**Figure 6:**
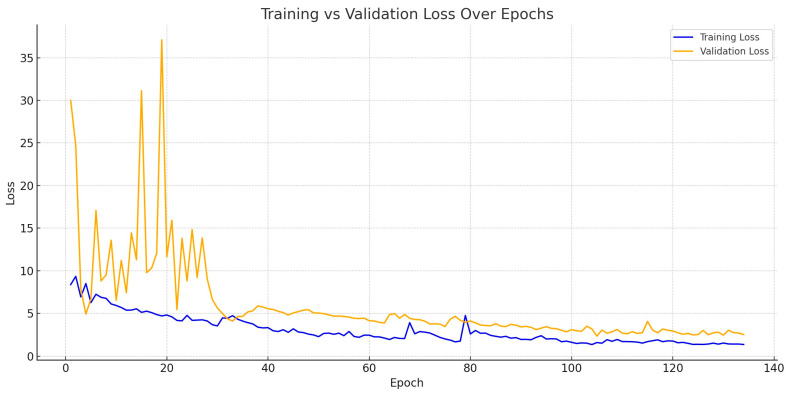
Training loss vs. validation loss progression of Mask R-CNN over 134 epochs.

The three models diverged in terms of training strategy, resource demands, and speed:

− YOLOv8 trained for 173 epochs, with 3.08 million parameters, achieving 0.913 mAP@0.5 and 0.821 mAP@[0.5:0.95] on training keypoints in just 0.78 h. Its average inference speed was 72–88 ms/image, yielding a strong ~13 FPS on CPU. The model stopped early using the Ultralytics framework’s default early stopping strategy, which monitors validation performance and halts training after 50 epochs without improvement.
− YOLOv11 completed 200 training epochs, using 2.65 million parameters across a deeper 109-layer network, and the best performance was recorded at epoch 138. Although it has lower GFLOPs (6.6 vs. 8.3 in YOLOv8), it achieved higher keypoint detection training accuracy: mAP@0.5 of 0.948 and mAP@[0.5:0.95] of 0.873. The evolution of mAP@0.5 and mAP@[0.5:0.95] during training is shown in Figure 4 and Figure 5 for YOLOv8 and YOLOv11, respectively. According to these two figures, there are observable differences in mAP between the pose detection and bounding box detection tasks in both YOLOv8 and YOLOv11 models. In YOLO’s architecture, these tasks are trained and evaluated independently: keypoint detection directly regresses spatial coordinates, whereas bounding box detection is optimized using Intersection-over-Union (IoU)-based objectives. This distinction can lead to performance differences, particularly when evaluated across varying IoU thresholds. The gap is more pronounced in the mAP@[0.5:0.95] metric shown in Figure 5, which incorporates increasingly strict IoU thresholds and thus magnifies sensitivity to small localization errors. As this study primarily focuses on pose estimation capabilities rather than traditional object detection, this convergence at lower thresholds further supports the robustness and reliability of our keypoint-based approach.
− Mask R-CNN had the highest parameter count (~59 million) and was trained and evaluated on a GPU, which enabled the use of full-resolution images (15,054 × 3214 px) without resizing. The model employed an early stopping strategy to prevent overfitting and reduce unnecessary computation. Specifically, training was halted after 30 consecutive epochs without improvement in validation loss, resulting in the model being saved at epoch 134, where it achieved the best performance. Despite the longer inference time of ~4.1 s per image (due to large input size and multi-task processing), Mask R-CNN delivered the highest performance in training keypoint detection metrics. The training convergence of Mask R-CNN is illustrated in Figure 6, which plots the model’s total loss over 134 epochs.
− Mask R-CNN had the highest parameter count (~59 million) and was trained and evaluated on a GPU, which enabled the use of full-resolution images (15,054 × 3214 px) without resizing. To prevent overfitting and reduce unnecessary computation, an early stopping strategy was employed, halting training after 30 consecutive epochs without improvement in validation loss. This resulted in the model being saved at epoch 134, where it achieved optimal performance. Despite a longer inference time of ~4.1 s per image (due to the large input size and multi-task architecture), Mask R-CNN achieved the highest performance in keypoint detection metrics. The progression of both training and validation losses over the 134 epochs is shown in Figure 6, providing evidence of stable convergence and supporting the effectiveness of the early stopping mechanism in avoiding overfitting. The apparent discrepancy between training and test keypoint recall in Table 2 is expected in small real-world datasets and does not necessarily indicate severe overfitting. This is supported by the training and validation loss curves, which show no divergence or stagnation.

#### 4.1.2. Models Performance on Test Data

The evaluation on the test dataset revealed strong performance across all three models in both bounding box and keypoint detection tasks. For bounding box detection, while YOLOv8 and YOLOv11 achieved higher precision, Mask R-CNN demonstrated the highest overall F1-score, offering balanced performance with strong recall (0.833) but slightly lower precision (0.773), indicating a tendency to include some false positives while capturing most true bounding boxes. However, as the primary focus of this research is on keypoint detection of hyperbolic reflections, the discussion below concentrates on keypoint-related performance metrics and trade-offs.

− YOLOv8 achieved the highest keypoint precision at 0.925, showing its strength in producing highly accurate predictions with minimal false positives. However, its recall was lower at 0.709, suggesting that it missed more true keypoints than the other models. This makes YOLOv8 particularly effective in contexts where precision is more important than completeness.
− YOLOv11, on the other hand, presented a more balanced performance with a precision of 0.817 and recall of 0.810, resulting in an F1-score of 0.814. This balance suggests that YOLOv11 is better suited for scenarios requiring both reliable detection and good coverage of true keypoints.
− Mask R-CNN outperformed both YOLO models in terms of F1-score, reaching 0.822, due to its combination of precision (0.811) and the highest recall (0.833) among the three. This indicates that Mask R-CNN was the most effective in capturing nearly all relevant keypoints while maintaining reasonable accuracy. Its performance benefited from operating on full-resolution images, which may have helped in better localizing spatially distributed summit points.

**Figure 7:**
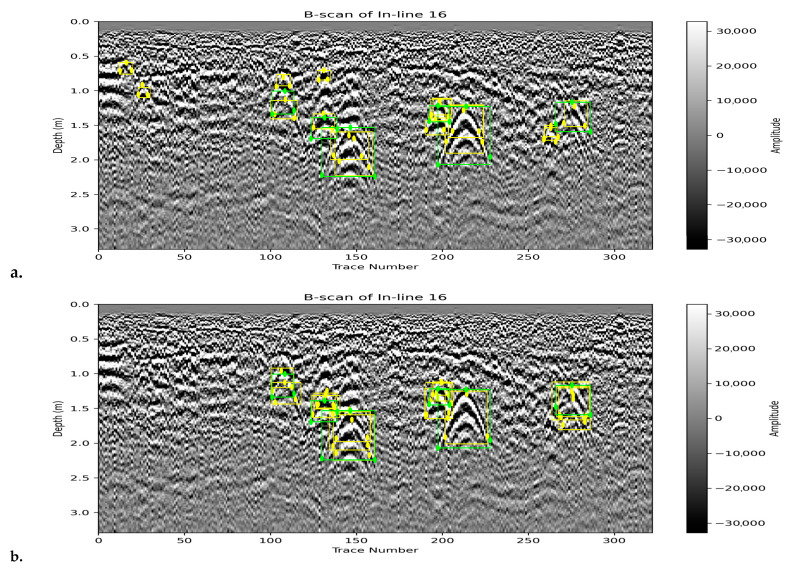

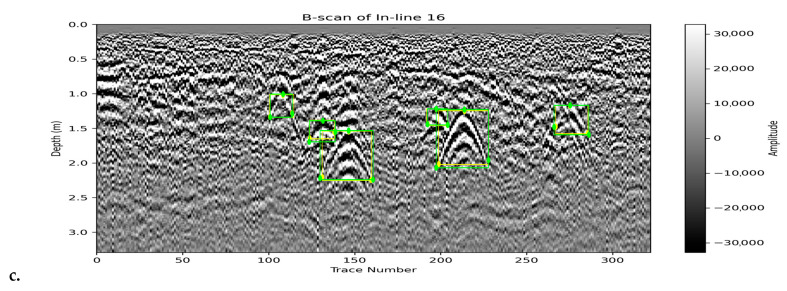
Sample visualization of bounding box and keypoint predictions (yellow) and ground truth annotations (green) across one of the test B-scans for all three models: (**a**). YOLOv8, (**b**). YOLOv11 and (**c**). Mask R-CNN.

In summary, YOLOv8 is best when high precision is essential, YOLOv11 provides balanced detection, and Mask R-CNN delivers the best overall keypoint detection performance in terms of F1-score on the test set. The visual comparison of predicted bounding boxes and keypoints (in yellow) against the ground truth annotations (in green) across a test B-scan is illustrated in Figure 7.

### 4.2. Clustering Detected Summit Points

Summit keypoints predicted by the Mask R-CNN model were used for subsequent 3D clustering to identify potential underground linear features. Summit keypoints predicted by the Mask R-CNN model, which achieved the highest pose recall and F1-score on both training and test datasets, were selected for subsequent 3D clustering and line fitting tasks. In our framework, the predictions undergo clustering using the DBSCAN algorithm, followed by line reconstruction via TLS and RANSAC. While RANSAC is robust to false positives, a detector with high recall and sufficient precision helps ensure that minimal true summit points are missed, especially when detections are sparse. Mask R-CNN offered this balance, with a test recall of 0.833 and precision of 0.811 for pose detection, thereby minimizing the risk of missing valid hyperbolas while maintaining relatively low noise. The clustering was performed using the DBSCAN algorithm applied to normalized coordinates representing trace number (X), sample-based depth (Z), and B-scan index converted to spatial position (Y). This enabled spatial grouping of keypoints in three dimensions, preserving the physical layout of the B-scans.

The DBSCAN parameters were tuned empirically to suit the sparsity and orientation of hyperbolic patterns typically observed in GPR data:

− eps = 0.5: defines the maximum distance between two points to be considered neighbors;
− min_samples = 5: sets the minimum number of keypoints required to form a dense region.

These parameters were empirically selected through iterative testing on the training data to balance noise reduction with preservation of continuous summit point clusters. We varied eps between 0.3 and 0.7 and observed the resulting cluster shapes and counts.

**Figure 8:**
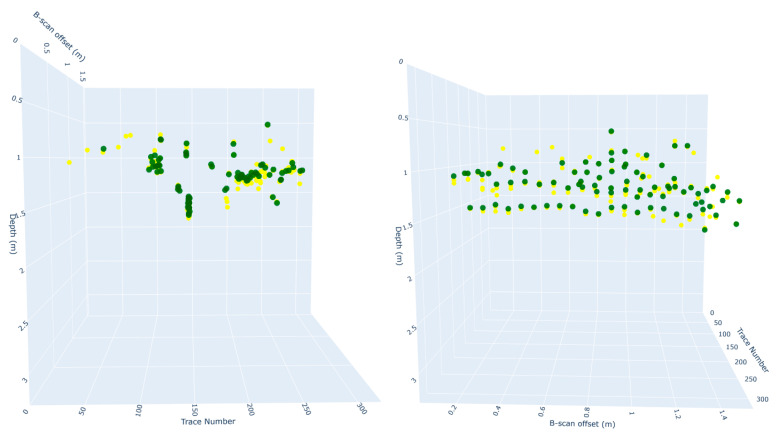
Three-dimensional visualization of all detected (yellow) and annotated (green) summit points in space from two different angles.

**Figure 9:**
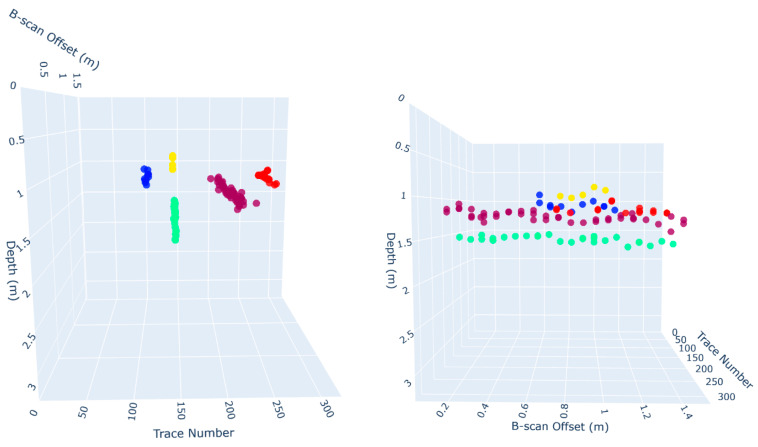
Three-dimensional scatter plot of clustered summit points based on eps = 0.5 and min_samples = 5.

Figure 8 provides a 3D visualization of all detected and annotated points. Two viewpoints are shown to help assess alignment, one along the trace number axis (X) and another along the B-scan position. Figure 9 also visualizes the selected output of the clustering step, showing color-coded summit point clusters that exhibit elongated spatial formations. The two perspectives again help highlight the spatial continuity and spread of each cluster. These visual patterns suggest the presence of subsurface structures and validate the potential of clustering to group discrete predictions into higher-order geometric interpretations.

**Figure 10:**
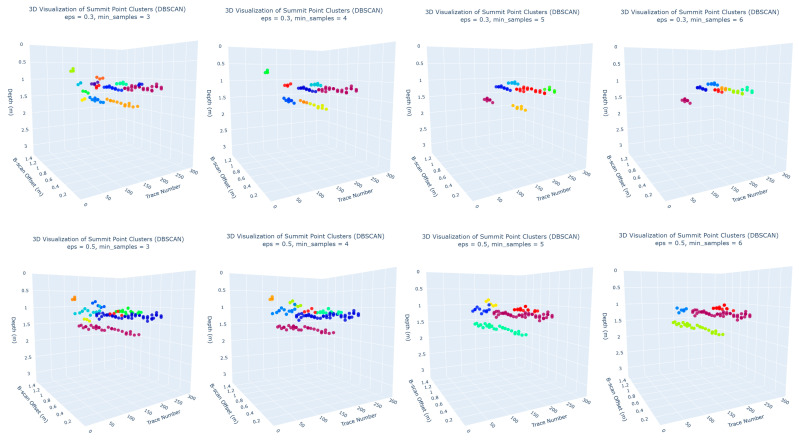

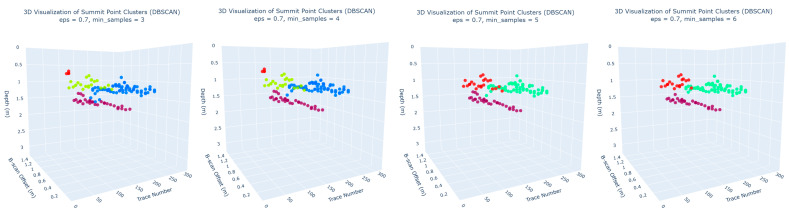
DBSCAN clustering outcomes under varying parameter settings.

As illustrated in Figure 10, the clustering results varied notably across different combinations of eps and min_samples. The setting of eps = 0.5 and min_samples = 5 visually achieved the best balance between outlier exclusion and accurate grouping of summit points into coherent linear clusters. This configuration avoided under-clustering (fragmenting a single utility line into multiple small clusters) and over-clustering (merging summit points from unrelated features), while also maintaining good noise control. These settings enabled the algorithm to distinguish linear aggregations from isolated noise. As a result, five major clusters were identified as candidate groups representing summit points potentially belonging to continuous underground features.

### 4.3. Fitting 3D Linear Models to Clusters

Following 3D clustering, each summit cluster was passed through a geometric fitting pipeline to reconstruct continuous 3D lines. Given the physical and technological limitations, detected summit points often form sparse and irregular distributions that do not directly trace the buried utility lines. Therefore, the objective was not to recreate a literal path but to generate an optimized simulation of hyperbolic reflections in 3D space.

Two models were evaluated. RANSAC as a robust estimator rejecting outliers while fitting a 3D line, and TLS as a linear subspace projection minimizing orthogonal errors. For line fitting using RANSAC, we used “RANSACRegressor” from “scikit-learn”. The following hyperparameters were employed:

− Maximum trials: 100 (i.e., the number of iterations);
− Minimum samples: default (determined automatically by the algorithm);
− Residual threshold: default (estimated based on the scale of the input data);
− Stop probability: 0.99;
− Loss function: absolute error.

These settings allowed the model to dynamically estimate an appropriate inlier threshold and robustly fit a line to summit keypoints within each cluster, without requiring manual tuning for different cluster sizes or noise levels.

To evaluate model fit, we calculated the RMSE for both TLS and RANSAC over all points in each cluster, not just inliers. This ensures a fair comparison, addressing concerns that RANSAC RMSE can appear artificially low if calculated only over inliers. Results are presented in Table 3. RANSAC outperformed TLS in most evaluated clusters with an average RMSE of 0.0559. This indicates that RANSAC provided a more reliable and accurate approximation of the linear trends within clustered summit points, particularly by suppressing the influence of noisy or misdetected points.

It should be highlighted that as there was no available ground truth for the actual utility line geometry, this comparison does not evaluate which method more accurately recovers the “true” line. Instead, RMSE is used here as a relative measure of how well each model fits the observed summit points within each cluster. This unsupervised evaluation thus optimizes for internal consistency, not external accuracy.

**Figure 11:**
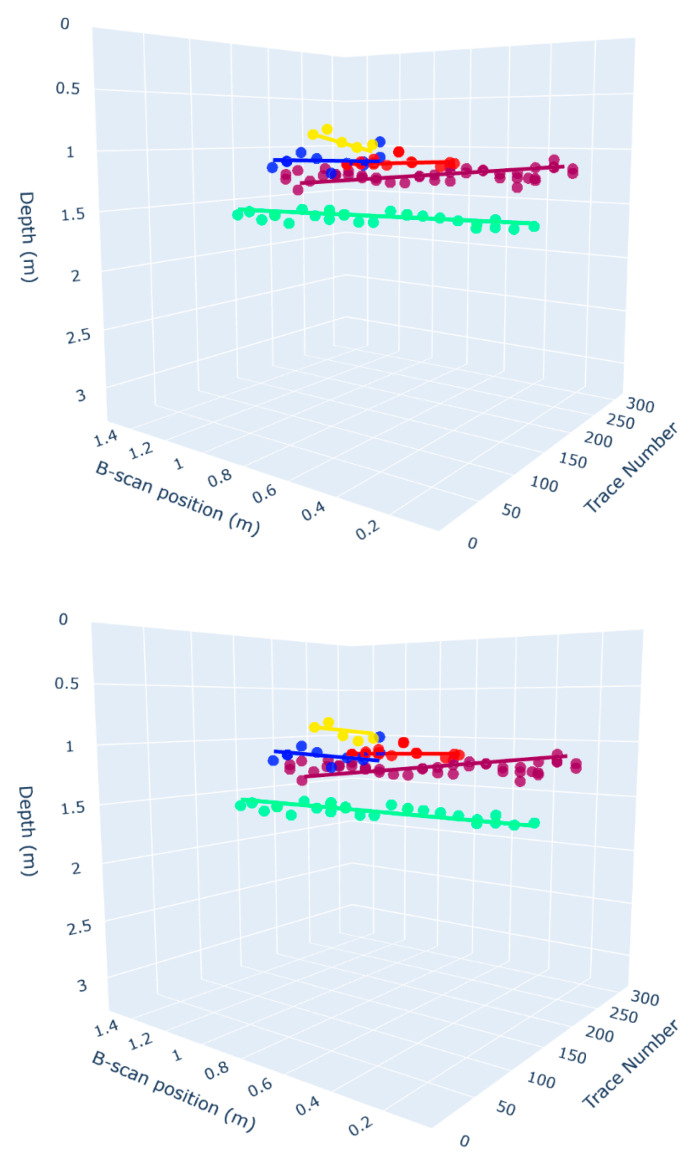
Three-dimensional visualization of fitted lines based on RANSAC (**top**) and TLS (**down**) overlaid on clustered summit points, showing linear trajectories that reflect buried infrastructure.

The outcome of this fitting process is illustrated in Figure 11, which presents a comparative 3D visualization of RANSAC-fitted lines (top) and TLS-fitted lines (bottom) overlaid on the clustered summit points. Both methods aim to extract coherent linear trajectories that plausibly represent underground utilities. While TLS provides a global fit sensitive to all data points, RANSAC demonstrates superior robustness to noise and outliers, yielding line segments that more consistently align with dominant summit clusters. This comparative visualization supports the choice of RANSAC for downstream trajectory reconstruction.

### 4.4. Methodological Discussion

As mentioned in Section 2.2 “Challenges in CNN-Based GPR Data Analysis”, despite the increasing use of deep learning for GPR interpretation, several persistent challenges continue to limit generalizability and field applicability. These include the scarcity of annotated real-world datasets, complex subsurface conditions, high false-positive rates and the difficulty of translating 2D detections into meaningful 3D representations. This study directly addresses these gaps through a hybrid and scalable pipeline that integrates object detection, keypoint estimation, 3D spatial clustering, and geometric modeling, anchored in high-resolution real-world data and expert-informed ground truth annotations.

Many recent studies, including [27], have highlighted the over-reliance on synthetic datasets due to the scarcity of annotated real-world GPR scans. While synthetic data and augmentation techniques such as GANs [26] help improve training diversity, they often introduce a domain gap that undermines generalizability. In contrast, our study was conducted using real-world GPR data, annotated under the guidance of an experienced geophysicist. This ensured that hyperbola boundaries and summit points reflect realistic geological conditions, making our output both practical and transferable to field conditions.

Recent studies [9,24,28,29], have attempted to address the inherent complexity of GPR data—marked by overlapping hyperbolic signatures and heterogeneous subsurface conditions—by employing 3D-CNNs and multi-scan fusion techniques. An example is Zhou et al. [47] who carried out direct volumetric learning on C-scans and full 3D blocks, which can leverage continuity and reduce cross-scan association errors. However, such methods typically presume dense, uniformly spaced acquisition, demand larger annotated volumes and substantially higher GPU/memory, and often struggle to generalize under irregular sampling or noisy soils. In contrast, our B-scan–centric pipeline bridges this gap by coupling pixel-level summit keypoints with 3D-DBSCAN clustering and RANSAC line fitting: it works with sparser/irregular line spacing, preserves the hyperbola interpretability familiar to practitioners, reduces labeling/compute burden, and allows incremental lines without re-volumizing—while still recovering 3D utility trajectories. The DBSCAN step grouped summit keypoints across multiple B-scans, simulating linear underground structures in 3D space without requiring complete 3D GPR volumes. DBSCAN was chosen for its ability to detect clusters of arbitrary shapes without the need to predefine the number of clusters, an advantage in scenarios where the number and continuity of utilities are unknown. Its robustness to noise and tolerance for irregular point densities make it well-suited for real-world GPR datasets, which often contain missing or weak reflections due to complex subsurface conditions. This method enabled a scalable and flexible approximation of underground utility trajectories, supporting reliable 3D reconstruction under practical field constraints.

GPR detection models often struggle with high false-positive rates due to artefacts from multiple reflections or complex soil conditions [22]. Our quantitative evaluation across both bounding box and keypoint metrics highlighted the architectural trade-offs between YOLO variants and Mask R-CNN. YOLOv8 achieved the highest precision (0.925) but lower recall (0.709), ideal for real-time inference in constrained environments where false positives must be minimized. In contrast, Mask R-CNN achieved the highest recall (0.833) and the best F1-score (0.822), making it more suitable for offline analysis where completeness is critical. The balanced performance of YOLOv11 also offers a practical trade-off for applications requiring both real-time response and broad coverage. These comparisons provide field-oriented guidelines on model selection based on task requirements.

Furthermore, the five reconstructed linear features shown in Figure 11 exhibit varied intersection angles between the GPR survey lines and the underlying utilities. This geometric diversity illustrates that the proposed hybrid deep learning and 3D geometric pipeline is robust to variations in intersection angle. Despite the known sensitivity of hyperbolic reflection geometry to the orientation of buried objects, the model consistently extracted coherent summit clusters and fitted accurate 3D trajectories using RANSAC. This outcome suggests that the combination of CNN-based keypoint detection and post-clustering fitting offers strong generalization across subsurface structures with diverse orientations.

A key methodological innovation of this study lies in its two-stage detection pipeline, which first localizes hyperbolic signatures using bounding box detection, and subsequently estimates their summit points (vertices) through keypoint detection. This approach enables a spatially precise representation of underground features, especially when combined with 3D clustering and geometric fitting, without relying on full volumetric GPR data. It is important to emphasize that in architectures such as YOLO and Mask R-CNN, keypoint detection is not an independent process; rather, it is inherently dependent on accurate bounding box localization. Keypoints are defined and predicted relative to these bounding boxes, and therefore, the detection of vertices is conditioned upon successful object detection. As such, it is not feasible, nor technically meaningful, to isolate and compare the models with and without keypoint detection as if they were independent modules. Instead, the innovation presented in this study lies in how keypoint information is integrated into a broader pipeline for 3D reconstruction, rather than in merely using keypoints as an auxiliary feature. This integration allowed us to approximate utility trajectories across B-scans without the need for full 3D GPR volumes, offering a lightweight, scalable alternative that balances precision and practicality under real-world constraints.

### 4.5. Study Implications

The findings from this study have both theoretical and practical implications. This research shifts the GPR hyperbola detection paradigm from 2D-only image segmentation to full 3D structure interpretation. The use of keypoint clustering and 3D line fitting introduces a new methodological pathway that can support spatially aware utility mapping systems—particularly relevant for civil engineering, archeology, and geotechnical investigations. By using real-world GPR data annotated with expert guidance, the study ensures its findings are applicable to actual field conditions. This increases the relevance and generalizability of the proposed method beyond synthetic or idealized datasets. This study offers a practical and adaptable framework that moves beyond hyperbola detection to hyperbola interpretation, delivering a reproducible pipeline for turning raw B-scan images into 3D spatial inferences about underground utilities, grounded in real-world data and expert-validated annotations.

## 5. Conclusions

This study presented a novel deep learning-based pipeline for detecting and spatially reconstructing linear underground utilities using GPR B-scan data. Focusing on the summit points of hyperbolic reflections, the framework combined object detection, keypoint estimation, 3D clustering and geometric fitting to simulate the trajectories of subsurface infrastructure in 3D space. We systematically compared the performance of YOLOv8, YOLOv11 and Mask R-CNN for both bounding box and keypoint detection, with Mask R-CNN demonstrating superior performance in keypoint recall and F1-score. Clustering of the predicted summit points via DBSCAN enabled spatial grouping of likely utility features, followed by RANSAC-based linear fitting to approximate plausible buried utility paths.

The scope of this work was explicitly focused on linear utilities (e.g., pipes or cables) that generate consistent hyperbolic patterns across multiple adjacent B-scans. While our method proved effective in approximating such features, a key limitation lies in the relatively small number of hyperbolas present in the dataset. We recognize that while real-world data improves field transferability, the relatively small number of B-scan slices limited both the training of the detection models and the robustness of post-detection clustering, where detection precision, rather than balanced F1-score, may be more critical due to the sensitivity of clustering algorithms to false positives. Similarly, the limited number of summit keypoints and resulting clusters constrained the ability to robustly validate or apply more sophisticated line-fitting methods beyond RANSAC. To address this, future work should focus on expanding the dataset with a richer variety of hyperbolas, both related and unrelated to linear utilities, collected under diverse subsurface and environmental conditions. This will allow us to explore model compression or knowledge distillation to improve generalization in resource-constrained settings. Additionally, increasing the number of accurately annotated summit points will enable the exploration of advanced 3D representation learning techniques such as PointNet/PointNet++, Graph Neural Networks (GNNs) or PointTransformer for the geometric modeling tasks.

Furthermore, while this work focused on detection and 3D reconstruction, the pipeline naturally extends to attribute-aware linear-utility classification (e.g., metal pipe, plastic conduit, cable). Standard GPR attributes—instantaneous/envelope amplitude and phase—and spectral descriptors (e.g., frequency centroid and bandwidth) can be computed in apex neighborhoods and fused either early (as additional channels for YOLO/Mask R-CNN) or late (concatenated with keypoint/cluster features). Following 3D-DBSCAN, trajectory-level summaries (e.g., median envelope, attenuation slope, phase stability) can feed a lightweight classifier to assign utility type. Implementing this extension requires verified labels (e.g., potholing/records) and is a priority for future work.

Overall, this study demonstrates the feasibility and value of integrating keypoint detection with spatial reasoning for underground utility mapping using real-world GPR data. The proposed approach offers a transparent and modular pipeline that enables interpretable and component-wise performance analysis. With further enhancements in data volume and annotation quality, it can be extended to capture more complex subsurface configurations through advanced geometric and spatial modeling techniques.

## Figures and Tables

**Table 1:** Model training and configuration summary.

Model	Image Size	Batch Size	Epochs (Early Stopping At)	Parameters	Layers	Optimizer	Learning Rate	Training Time
YOLOv8	640 × 640	8	200 (173)	3.08 M	81	SGD	0.01	0.78 h
YOLOv11	640 × 640	8	200	2.65 M	109	SGD	0.01	0.85 h
Mask R-CNN	15,054 × 3214	8	200 (134)	59 M	101	Adam	0.001	~8.5 h

**Table 2:** Detection and keypoint estimation metrics.

Dataset	Model	Pose Precision	Pose Recall	Pose F1	Box Precision	Box Recall	Box F1
Train	YOLOv8	0.802	0.833	0.818	1	0.647	0.785
	YOLOv11	0.851	0.957	0.901	0.73	1	0.844
	Mask R-CNN	**0.897**	**1**	**0.946**	0.885	0.986	0.932
Test	YOLOv8	**0.925**	0.709	0.803	0.946	0.663	0.78
	YOLOv11	0.817	0.81	0.814	0.821	0.754	0.786
	Mask R-CNN	0.811	**0.833**	**0.822**	0.773	0.833	0.867

**Table 3:** RMSE Comparison of Line Fitting Models.

Cluster ID	Points in Cluster	RANSAC RMSE	TLS RMSE	Best Method
0	21	0.1312	0.1602	RANSAC
1	4	0.0287	0.0347	RANSAC
2	18	0.0305	0.1335	RANSAC
3	6	0.0613	0.0577	TLS
4	4	0.0279	0.0290	RANSAC
Avg.		0.0559	0.0830	RANSAC

## Data Availability

The authors do not have permission to share data. The GPR dataset used in this study was provided under a restricted agreement. However, the full experimental pipeline, including model architecture, training configurations, and evaluation metrics, has been described in detail to support reproducibility. Researchers interested in further details may contact the corresponding author for guidance on applying the pipeline to alternative datasets.
